# Coding or Noncoding, the Converging Concepts of RNAs

**DOI:** 10.3389/fgene.2019.00496

**Published:** 2019-05-22

**Authors:** Jing Li, Changning Liu

**Affiliations:** CAS Key Laboratory of Tropical Plant Resource and Sustainable Use, Xishuangbanna Tropical Botanical Garden, The Innovative Academy of Seed Design, Chinese Academy of Sciences, Kunming, China

**Keywords:** messenger RNA, long noncoding RNA, coding potential, ribosome profiling, micropeptide

## Abstract

Technological advances over the past decade have unraveled the remarkable complexity of RNA. The identification of small peptides encoded by long non-coding RNAs (lncRNAs) as well as regulatory functions mediated by non-coding regions of mRNAs have further complicated our understanding of the multifaceted functions of RNA. In this review, we summarize current evidence pointing to dual roles of RNA molecules defined by their coding and non-coding potentials. We also discuss how the emerging roles of RNA transform our understanding of gene expression and evolution.

## Introduction

Benefiting from the advances in science and technology, our understanding of the complexity of organisms is constantly increasing. The “central dogma” of molecular biology states that genetic information is typically processed from DNA to RNA to protein, and this decides cellular and organismal phenotype (Crick, 1970). In the past, RNAs, except for infrastructural RNAs (such as rRNAs and tRNAs), were commonly considered as an intermediate between DNA and proteins. However, over recent decades, the rapid development of high-throughput sequencing technologies has revealed the pervasive transcription of eukaryotic genomes (Okazaki et al., 2002; Carninci et al., 2005; Kapranov et al., 2007; Lander, 2011), thus revealing RNA-mediated gene regulation. The fact that most regulatory RNAs function without involvement in protein translation led us to re-examine the roles of RNAs in the development and evolution of higher organisms.

In higher organisms, only a small fraction of genetic transcripts (less than 3%) have the capability to encode proteins, despite pervasive transcription across genomes. This raises the question of whether the remaining non-protein-coding transcripts are transcriptional “noise” or contain more genetic information. Large-scale projects for the systematic annotation and functional characterization of genes (such as ENCODE and FANTOM) have reported that at least 80% of mammalian genomic DNA is actively transcribed and elaborately regulated, with the vast majority of this considered to be noncoding RNA (ncRNA) genes (Consortium, 2012; Hon et al., 2017). The numbers of ncRNA genes vary between species, and interestingly, the complexity of an organism is highly associated with the abundance of ncRNA genes but not protein-coding genes, implying the potential importance of ncRNAs (Rubin et al., 2000; Stover et al., 2000; Mattick, 2001; Venter et al., 2001; Kapusta and Feschotte, 2014). Among these, lncRNAs that are defined as transcripts longer than 200 nucleotides with low/no protein-coding potential, represent a considerable proportion.

Long non-coding RNAs can regulate gene expression in various ways, including epigenetic, transcriptional, post-transcriptional, translational and protein location effects. Corresponding to functional diversity, the modes of action of lncRNAs are also quite varied. lncRNAs can recruit epigenetic factors to modify chromatin state (Rinn and Chang, 2012), assemble transcriptional machinery to trigger the initiation of transcription (Bonasio and Shiekhattar, 2014), or act as a structural organizer to participate in the formation of subcellular organelles (Naganuma and Hirose, 2013). Additionally, lncRNAs can complementarily bind with other forms of RNA molecules to modulate gene expression at transcriptional, post-transcriptional and translational levels, for example as a moderator of mRNA activity or a decoy/sponge for miRNA (Poliseno et al., 2010; Gong and Maquat, 2011; Bonasio and Shiekhattar, 2014; Tay et al., 2014; Yoon et al., 2014). Moreover, lncRNAs couple with proteins through particular structures to act as a location transferor, or to modulate enzyme activities (Wang and Chang, 2011).

**FIGURE 1:**
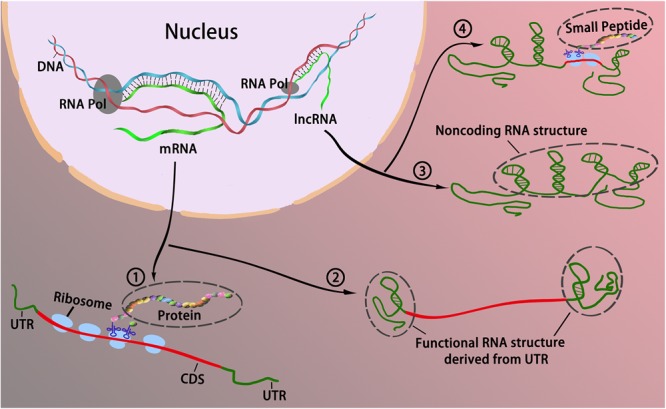
The interchangeable roles between coding and long noncoding RNAs. Traditionally, RNAs could be divided into two categories in accordance with their coding potential, that is, coding RNAs and noncoding RNAs. Coding RNAs generally refers to mRNA that encodes protein ① to act as various components including enzymes, cell structures, and signal transductors. Noncoding RNAs act as cellular regulators without encoding proteins ③. However, it appears that the boundaries blur between coding RNA and noncoding RNA as some coding mRNAs can function without translating to protein via the formation of RNA secondary structure primarily derived from the UTR ②; some lncRNAs can bind with ribosomes, and encode peptides to modulate cellular activities ④.

Based on the “noncoding” definition, the modes of action of lncRNAs mentioned above are exerted primarily through ncRNAs. Intriguingly, recent bioinformatics analyses of large-scale data from ribosome-protected RNA fragments (ribosome profiling or ribo-profiling) have revealed that a considerably large part of these transcripts tends to contain sORFs and binds with ribosomes (Aspden et al., 2014; Ruiz-Orera et al., 2014; Anderson et al., 2015; Mackowiak et al., 2015; Olexiouk et al., 2016), suggesting that the coding potential of lncRNAs has been vastly underestimated. Several functional experiments have demonstrated that some lncRNAs can encode small peptides (named “micropeptides” with a length less than 100 aa) that are involved in various biological processes, although this is rare (Hubé and Francastel, 2018). In addition, certain coding transcripts, such as TP53 mRNA, could also function as RNA, without translation to proteins, to regulate significant biological processes (Candeias, 2011; Kloc et al., 2011). Therefore, it seems reasonable to presume that the demarcation of RNA depending on its coding or noncoding status is somewhat blurred, and partially intertwined. That is, RNA roles are likely not tightly constrained (such as RNA functioning only as mRNA or ncRNA), but rather converge and overlap: lncRNAs can function by encoding small peptides, while mRNAs can use their special structural features, such as the 3′ UTR or 5′ UTR, to function (Figure 1).

In the present article, we will review current studies of the bilateral functionality of lncRNAs and mRNAs in terms of their coding potential, as well as the advancement of high-throughput techniques that would facilitate a deeper recognition of functional diversity of RNAs. This review will highlight the cases that illuminate the contrapositive roles between lncRNAs and mRNAs, and briefly discuss the biological significance of these discoveries for gene expression and evolution.

## Long Noncoding Rnas Encode Small Peptides/Proteins With Regulatory Functions

### Peptides/Proteins Encoded by Regular Long Noncoding RNAs

The original definition of lncRNAs concerns their low/non-coding potential. However, with accumulating evidence from bioinformatics and ribosome transcriptome profiling, lncRNAs have been shown to display strong ribosomal associations in many species, varying from plant to animal, indicating a potential coding capacity in lncRNA sORFs (Kageyama et al., 2011; Nam et al., 2016; Yeasmin et al., 2018). In recent years, several micropeptides derived from lncRNAs have been shown to be functional. We have summarized these micropeptides in Table 1.

**Table 1:** Peptides encoded by lncRNAs in plants and animals.

Origin	Micropeptides	Gene	Function	Size (AAs)	References
Plants	Early nodulin 40 (Enod 40)	*Enod 40*	Nodule organogenesis	12; 24	Rohrig et al., 2002
Plants	POLARIS (PLS)	*POLARIS*	Leaf morphogenesis	36	Chilley et al., 2006
Plants	ROTUNDIFOLIA (ROT4)	*ROT4*	Leaf morphogenesis	53	Narita et al., 2004
Plants	ROT18/ DLV1	*DEVIL1 (DVL1)*	Plant organogenesis	51	Wen et al., 2004
Plants	Kiss of death (KOD)	*Kiss of death (KOD)*	Programmed cell death regulation	25	Blanvillain et al., 2011
Plants and animals	Brick1 (Brk)	*Brick1 (Brk)*	Leaf morphogenesis	84	Frank and Smith, 2002
Poaceae	Zm401p10; Zm908p11	*Zm401*	Pollen development	89; 97	Ma et al., 2008
Mammals	DWORF	*LOC100507537*	Enhance muscle performance	34	Nelson et al., 2016
Vertebrates	Toddler	*LOC100506013*	Promotes cell migration; activator of APJ/Apelin receptor signaling	54	Pauli et al., 2014
Vertebrates	Myomixer	*LOC101929726*	Functionally involve in controlling muscle performance	84	Bi et al., 2017
Fruit fly	Pri	*polished rice (pri)*	Epidermal morphogenesis in embryogenesis	11; 32	Kondo et al., 2007
Fruit fly	MOTS-c	*12s rRNA*	Insulin sensitivity and metabolic Homeostasis.	16	Lee et al., 2015
Fruit fly	Pgc polypeptide	*pgc*	Positivephosphorylation of transcription elongation factor b (P-TEFb)	71	Hanyu-Nakamura et al., 2008
Fruit fly	sarcolamban (Scl)	*pncr003:2L*	Calcium transport and muscle contraction,	28; 29	Magny et al., 2013
Fruit Fly	Tarsal-less/tal	*LP10384*	Morphogenesis, including tissue morphogenesis and pattern formation	11	Galindo et al., 2007
Mammals	PINT87aa	*LINC-PINT*	Inhibit the transcriptional elongation of multiple oncogenes	87	Zhang et al., 2018b
Mammals	Mitoregulin (Mtln)	*LINC00116*	Mitochondrial respiration, ROS, and Ca2+ retention capacity	56	Stein et al., 2018
Mammals	MRI-2	*C7orf49*	Non-homologous end-joining DNA repair	69	Slavoff et al., 2014
From humans to zebrafish	AGD3	*AGU1*	Human stem Cell differentiation	63	Kikuchi et al., 2009
Mammals	NoBody	*LINC01420/ LOC550643*	mRNA turnover and nonsense-mediated decay (NMD)	67	D’Lima et al., 2017
Mammals	Minion	*LOC101929726*	Muscle development	84	Zhang et al., 2017
Human and mouse	SPAR	*LINC00961*	Regulate muscle regeneration	90	Matsumoto et al., 2017
Primates	HOXB-AS3 peptide	*HOXB-AS3*	Suppresses colon cancer growth, PKM splicing and subsequent metabolic reprogramming	53	Huang et al., 2017
Different species	Humanin	*16s rRNA*	Program cell death	24	Hashimoto et al., 2001
Human	SRAP	*SRA*	Diverse roles in both normal biological processes and pathological changes	224; 236	Emberley et al., 2003; Chooniedass-Kothari et al., 2006
Mouse and human	Myoregulin (MLN)	*LINC00948* in human; *2310015B20Rik* in mouse	Inhibit the SERCA activity and regulate Ca^2+^ flow in muscle	46	Anderson et al., 2015

Steroid receptor RNA activator is a prototypic example of lncRNAs with both coding and noncoding products (Lanz et al., 1999; Mattick, 2003; Hubé et al., 2006, 2011). *SRA* was initially identified as a noncoding gene with multiple RNA isoforms, which is critical in many biological processes, such as acting as a co-activator of nuclear receptors and a regulator of steroid receptor-dependent gene expression (Hubé et al., 2006, 2011; Cooper et al., 2011). Interestingly, *SRA* can also encode for a conserved SRAP, which, in turn, represses the transcriptional regulatory activity of the *SRA1* gene by interacting with a specific SRA stem-loop (Emberley et al., 2003; Chooniedass-Kothari et al., 2006; Hubé et al., 2011). The transmissible functionalities between the coding and noncoding *SRA* gene are caused by alternative splicing (AS) of introns/extrons (Colley and Leedman, 2011), suggesting the significance of AS events in the generation of bifunctional RNA.

Of note, among the small number of already-known functional micropeptides, a few are muscle-specific, and have been implicated in the regulation of the activities of SERCA (Anderson et al., 2016; Nelson et al., 2016; Matsumoto et al., 2017). For example, MLN, a 46-aa micropeptide specifically expressed in skeletal-muscle, is encoded by a lncRNA (*LINC00948* in human and *2310015B20Rik* in mouse); it can directly interact with SERCA to decrease the affinity of this ATPase for Ca^2+^ and inhibit Ca^2+^ entry into the SR (Anderson et al., 2015). The Scl micropeptide is encoded by the noncoding *pncr003:2L* gene, and can affect Ca^2+^ traffic in cardiac muscle in the fly; the mutation of this gene triggers an arrhythmic phenotype (Magny et al., 2013). The MOTS-C micropeptide can regulate insulin sensitivity and metabolic homeostasis in the mitochondria of muscle cells, and derives from mitochondrial 12S rRNA (Slavoff et al., 2014; Lee et al., 2015). In the above example, the Scl peptides and their respective regulatory functions in the heart are quite conserved between species, including the fly and humans (Magny et al., 2013). These results indicate that several sORFs embedded in the noncoding region of the genome seem to undergo a relatively stricter natural selection than adjacent sequences, raising the question of whether these sORFs have a capability to sprout into a new gene *in situ* or to be integrated as a component into new genes elsewhere during evolution.

The *tal* gene in *Drosophila* is of vital importance in tarsal morphogenesis in the fly leg, and stage-and position-specific expression have been reported in embryonic development. Although *tal* is regarded as a noncoding gene as none of its ORFs are over 100 aa, deeper analysis has found that the functionality of *tal* is predominantly dependent on the ORF regions (Manak et al., 2006). There are five ORFs in the *tal* gene, four of which contain a similar and conserved 7 aa motif that determines the functionality of the gene, with the shortest peptide of only 11 aa. Phylogenetic analysis revealed that these *tal*-like peptides are conserved in metazoans and represent a new class of eukaryotic genes. The discovery of these mini-peptides further expands the possible scope and function of lncRNA-encoded peptides that are hidden in currently sequenced genomes and the transcriptome (Galindo et al., 2007).

### Peptides/Proteins Encoded by Circular RNAs

Circular RNAs pertain to a sub-category of specialized lncRNAs, which are primarily produced by backsplicing the 3′ end to the 5′ end of exons in the same transcript (often a coding gene) via the spliceosome, thereby forming lncRNAs in a circular shape (Ashwal-Fluss et al., 2014; Zhang X.O. et al., 2014; Starke et al., 2015). Through bioinformatics analysis and high-throughput sequencing, many circRNAs have been identified in multiple species (Sanger et al., 1976; Capel et al., 1993; Danan et al., 2012; Memczak et al., 2013; Jeck and Sharpless, 2014; Wang et al., 2014). However, understanding of circRNA function is still very limited. The reported biological activities of circRNAs include acting as a sponge for microRNAs (Hansen et al., 2013; Memczak et al., 2013), as a competitor during pre-mRNA splicing (Ashwal-Fluss et al., 2014), and as a transcriptional regulator in the nucleus (Li et al., 2015). The majority of circRNAs are chimeric lncRNAs derived from mRNA transcripts and likely in part encompass the exons of protein-coding genes. This poses the question of whether circRNAs have protein coding capabilities. In fact, many studies have demonstrated that circRNAs have coding capabilities both *in vitro* and *in vivo* in terms of cap-independent translation (Chen and Sarnow, 1995; Li and Lytton, 1999; Guo et al., 2014; Jeck and Sharpless, 2014; Abe et al., 2015; Wang and Wang, 2015; Pamudurti et al., 2017). Moreover, some functional protein products are encoded by circRNAs (such as *circ-FBXW7; circ-Mbl, circ-ZNF609* and *circ-SHPRH*) (Rybak-Wolf et al., 2015; Pamudurti et al., 2017; Yang et al., 2018; Zhang et al., 2018a).

*circ-ZNF609* was initially screened out in a functional genetic screen, and is differentially expressed during myogenesis (Legnini et al., 2017). This circRNA contains an ORF covering almost all ORF regions of the host gene, but has a small variation at the splice junction. Its protein product lacks the zinc-finger domain compared with its linear counterpart, with an obvious impact on myoblast proliferation. Interestingly, heat shock could significantly activate the translation of *circ-ZNF609;* suggesting a possible regulatory role of circRNA translation under specific stimuli (Legnini et al., 2017).

*circ-Mbl* was first detected in the lodge of the second exon of the splicing factor muscleblind (MBL/MBNL1) in flies and humans, with a function of competing with pre-mRNA splicing (Ashwal-Fluss et al., 2014). Recently, through a bioinformatics analysis of ribosome foot-printing datasets, Pamudurti and co-workers revealed that *circ-Mbl* could encode a peptide in the fly head, as detected through MS. Both *circ-Mbl1* RNA and its protein-related product reside in the synaptosome and can be regulated by the 4E-BP and the transcription factor in forkhead family – FOXO, suggesting that this circRNA translation might be distinctively important in the brain (Pamudurti et al., 2017).

The observation that circRNAs generate proteins can be traced back to much earlier studies in *Archaea*, where circularized introns produce a site-specific endonuclease (Dalgaard et al., 1993). However, to date, direct experimental evidence for circRNA translation to peptides is still scarce; as a result, it is even tougher to understand the function of their translated products. Considering that most circRNAs stem from coding transcripts and contain complete exons, it is possibly assumed that the circRNAs and their coding-products might provide uncharacterized modes of regulation of gene and protein expression (Pamudurti et al., 2017). Therefore, it is important to further investigate the possible functions associated with circRNA coding.

### Large-Scale Approaches for the Identification of Potential sORFs

To date, hundreds of thousands of lncRNAs have been discovered in various species, and there is a desire to study their relevant functional mechanisms (Okazaki et al., 2002; Liu et al., 2005; Kapranov et al., 2007; Ponting et al., 2009; Ulitsky and Bartel, 2013; Volders et al., 2013). However, it is unpractical to identify lncRNAs and predict their functions using only traditional technical approaches, irrespective of the requirement for intensive validation of the exact mechanisms underlying lncRNA activities. The same is true for the identification of lncRNA coding capacity. Therefore, new large-scale technologic approaches based on computational analysis of transcriptome data and proteomics data have been developed, all of which are mutually reinforcing and cross-validated.

A ribo-seq technique has been recently developed and is widely used to measure the full coding potential of RNA transcripts on a genome scale through deep sequencing of ribosome-protected RNA fragments (Ingolia et al., 2009). By identifying the precise ribosomal positions of RNAs, ribo-seq can plot the potential on-going events of translation in the cytosol, which is useful in identifying potentially functional micropeptides (Ingolia et al., 2011; Ingolia, 2016). With the advent of Ribo-Seq, thousands of translated sORFs were discovered in lncRNAs (Ingolia et al., 2011; Bazzini et al., 2014; Ruiz-Orera et al., 2014; Ji et al., 2015), with a few functional peptides, such as MLN (Anderson et al., 2015) and HOXB-AS3 (Huang et al., 2017). However, the proportion of coding lncRNAs estimated by various ribosome-profiling studies differ widely (Guttman et al., 2013; Ingolia et al., 2014), resulting from false positive and distinct prediction thresholds. Therefore, MS has emerged as a complementary method.

Mass spectrometry demonstrates excellent performance in detecting and characterizing the products of proteins/peptides in a complex biological sample. The detection of lncRNA-encoded peptides is the most direct evidence for lncRNA coding potential. However, to date, the proportion of coding lncRNAs detected by MS-based proteomes is small compared with that in ribo-seq results (Verheggen et al., 2017). The main weakness attributed to this approach is that MS-based proteomics is obviously impacted by the length and concentration of the detected samples. Therefore, specialized methods have been developed to circumvent these detection limitations. Short translation products at low abundance can surmount the threshold of MS detection through the use of peptidomics approaches (Schulz-Knappe et al., 2005) and enrichment protocols (Mustafa et al., 2015).

Both of the above techniques have their respective advantages and shortcomings; therefore, “proteogenomics” has been developed (Nesvizhskii, 2014; Menschaert and Fenyö, 2017; Ruggles et al., 2017). In proteogenomics, proteomics data are systematically integrated and analyzed with genomics and transcriptomic data generated from DNA-sequencing, RNA-sequencing and ribosome-profiling. The predicted sequences of proteins/peptides are tracked back to the genome and transcripts to identify the gene expression patterns and actual translational events. The significance of proteogenomics studies lies in improving genome annotation, and reasonably applying multi-omics data to explore complex and profound mechanisms in biological activities and complex diseases (Zhang B. et al., 2014; Zhang et al., 2016; Mertins et al., 2016).

## Noncoding RNA Regulatory Functions Embedded in mRNAs

### 3′ UTR Regulatory Roles of mRNAs

Based on current research results, the noncoding regulatory functions discovered in mRNAs are mainly present in the 3′ UTRs, which were previously supposed to be the vital regulative elements for mRNA self-stability and location. Compared with highly conserved coding regions that have to undergo strictly selected pressure, the 3′ UTR displays more flexibility and plasticity between species. Its size varies from a few to hundreds of nucleotides, and likely has a close relationship with biological complexity (Chen et al., 2012; Mayr, 2016). Moreover, for an RNA molecule, other than the impact on base pairing, the changes in sequence are most likely to induce corresponding changes in structure, resulting in information transmitted from RNA to protein (Berkovits and Mayr, 2015).

By comprehensively estimating up-to-date cases where mRNAs regulate biologic activities without translating to protein, we found that the 3′ UTR of mRNA plays a large role as an effectors. Increasing evidence has demonstrated that the 3′ UTRs of mRNAs are actively involved in repressing the occurrence and progression of cancer cells, such as the 3′ UTRs of α*-tropomyosin* mRNA, *prohibitin* mRNA and *ribonucleotide reductase* mRNA (Rastinejad et al., 1993; Fan et al., 1996; Manjeshwar et al., 2003). These studies demonstrate that the 3′ UTRs of some mRNAs can antagonize tumor development, likely through RNA interactions with regulatory factors involved in cellular growth in a post-transcriptional pattern. Indeed, the 3′ UTR can recruit RNA-binding proteins, as in the case of *CD47* mRNA. *CD47* mRNA has two isoforms of the 3′ UTR, long (*CD47-LU*) and short (*CD47-SU*), and only the *CD47-LU*, which is AU-rich, can interact with the RNA-binding protein TIS11B to form a membraneless organelle with a specific biochemical and biophysical environment which is separate from the cytosol (Ma and Mayr, 2018). However, the most prevalent mode of action of the 3′ UTR is as ceRNAs, such as in the cases of *CCR2* mRNA and *Ube3a1* RNA, which confer to the function of lncRNAs (Valluy et al., 2015; Hu et al., 2017).

### Noncoding Regulatory Roles of mRNA Not Involving the 3′ UTR

Other than the 3′ UTR, the 5′ UTR and ORF can also be involved in RNA-mediated regulatory function, although recent reports of this phenomenon are scare. Two mRNAs, *TP53* mRNA and *HIST1H1C* mRNA, are recognized as being involved in ORF-mediated regulation. TP53 protein is a tumor suppressor implicated in many processes during tumor occurrence and development. However, a triple synonymous mutant (TriMp53) in codons led to a misshapen structure, resulting in loss of the IRES activity of p47 (one isoform of p53) and an abrogated affinity of hnRNPC, but with better binding to Mdm2, which is an E3 ubiquitin-protein ligase in mediating p53/TP53 ubiquitination, and an augmented ability of p53 to activate apoptosis. These facts indicate that TP53 has intricate regulatory roles at both the RNA and protein levels, suggesting that the functions of the RNA and protein molecules are closely intertwined (Candeias, 2011). *HIST1H1C* mRNA participates in regulating telomere length homeostasis. Aside from the protein-related product, a 15-nt long region in the ORF region (nt334–nt348) is attributed to *HIST1H1C*-mRNA-mediated biological activity, through complementation with the terminal stem-loop sequence of the P6b region of hTR, in a base-pairing pattern. These results extend the functional potency of mRNA ORF regions in a non-traditional and noncoding direction (Ivanyi-Nagy et al., 2018).

In terms of the 5′ UTR, there are only two examples. VEGF is a key regulator of angiogenesis during embryonal and cancerous development, and this regulatory function is closely correlated with the 5′ UTR. *vegf* mRNA has an unusually long 5′ UTR of 1,038 nucleotides, and contains two IRES, resulting in an intricate regulation of VEGF expression. In addition, the presence of the 5′ UTR of *vegf* mRNA alone in tumor cells could promote the expression of anti-apoptotic genes but repress pro-apoptotic genes, suggesting an anti-apoptotic role of the *vegf* 5′ UTR, and demonstrating its potential as a target for cancer treatment. To the best of our knowledge, the 5′ UTR of *vegf* mRNA represents the only example of an mRNA UTR which can promote tumor progression (Akiri et al., 1998; Huez et al., 1998; Masuda et al., 2008).

The *c-myc P0* transcript is an isoform transcript from the promoter 0 (P0) of the c-myc gene, which has an extra ˜639-nucleotide extension of the 5′ UTR when compared with two major isoforms (P1 and P2) of *c-myc* mRNA. Ectopic expression of the 5′ UTR of the *c-myc P0* transcript alone in HeLa cells results in significantly increased expression of the c-Myc1 (p67) and c-Myc2 (p64) proteins as well as incremental apoptosis sensitivity, but decreased tumorigenicity, all of which are likely attributable to competitive regulation of gene expression in the c-myc locus. These results demonstrate that the 5′ UTR potentially functions *in trans* to perform gene regulation (Blume et al., 2003).

## Perspective

In recent years, researchers have begun to pay close attention to the development of bifunctional RNAs, and have discussed the evolved roles of RNAs with multiple functions (Dinger et al., 2011; Ulveling et al., 2011; Kageyama et al., 2011; Hubé and Francastel, 2018). In the early stages of such research, researchers discovered individual gene on a case-by-case basis. However, in the last ten years, rapid advances in large-scale detection and identification techniques (such as ribo-seq and MS-based proteomics) have facilitated multi-faceted investigations of genomes and vital processes, thus shedding light on the complex activities of various RNA molecules. Bifunctional RNAs raise questions about the concept of a gene, in terms of whether RNA, both coding and noncoding, is an independent gene type or a convergence of coding and noncoding genes which occurred during evolution. In this review, we intend to not only investigate the current status of bifunctional RNAs as reported in recent years, but also discuss the potential pervasiveness of bifunctional RNAs from a global perspective in terms of large-scale data.

With recent estimates of ribosome profiling, small peptides encoded by lncRNAs have significantly expanded the extent and diversity of the proteome, and predictions suggested that a large fraction of the annotated lncRNAs in various eukaryotic organisms would be translated with sORFs (Aspden et al., 2014; Ruiz-Orera et al., 2014; Mackowiak et al., 2015; Olexiouk et al., 2016). Proteogenomic evidence has confirmed that many small peptides which stem from regions of lncRNA genes are expressed differentially in different cell types and during different developmental/disease stages, although their functions are somewhat enigmatic (Nesvizhskii, 2014; Zhu et al., 2018). However, other studies have revealed that mRNAs could also be involved in cellular regulatory processes in a coding-independent manner (Nam et al., 2016). The results from a large-scale RNA structure analysis revealed that the secondary structures of mRNA have an essential regulatory effect on its maturation and stability, even for the evolutionarily conserved RNA silencing pathways of eukaryotes, suggesting that mRNAs partially retain the functionality of structure that exists in many RNA molecules (Katz and Burge, 2003; Li et al., 2012; Taggart et al., 2012). All these facts indicate that the coding potential and biological roles of mRNAs and lncRNAs could be switched in some cases, implying a conceptual blurring between coding and noncoding genes.

Many lncRNAs share similar features with classical mRNAs, such as transcription by polymerase II with a 5′-cap and 3′-polyadenylated tail, and frequent accumulation in the cytoplasm (van Heesch et al., 2014). Therefore, when associated with ribosomes, sORFs embedded in lncRNAs have a significant chance to be translated to peptides. The peptides derived from lncRNAs have a relatively shorter chain length and weaker conservation across different species, and this is consistent with the original lncRNAs which often have few introns, a low expression level and weak phylogenetic conservation (Cabili et al., 2011; Derrien et al., 2012; Kutter et al., 2012; Necsulea et al., 2014). From the perspective of proteins driving evolution, these peptides are likely considered to be an important source for new protein (Ruiz-Orera et al., 2014). Previously reported experimental evidence indicates that noncoding RNAs expressed at low levels could contribute to the birth of novel protein coding genes (Levine et al., 2006; Cai et al., 2008; Reinhardt et al., 2013). Given that several lncRNA-derived peptides have been demonstrated to play essential roles in many biological activities, it is worth investigating the putative significance of the generation of these lncRNA-derived peptides in gene evolution, expression and regulation.

However, in view of the huge quantity, diversified mechanisms of action, and intricate functions of lncRNAs, it is inappropriate to regard lncRNAs just as a pool for evolved peptides. In terms of RNA alone, its roles are diverse, including the potential to be retro-transcribed into DNA, or to act as an enzyme to participate in complex biochemical processes (Cech, 1986). Moreover, random RNA sequences can inoculate structurally complex and highly active RNA ligases, suggesting that randomness can produce functionality (Ekland et al., 1995). Therefore, it is very likely that RNA molecules alone comprise abundant genetic information, such as particular structural features and ultra-conservative sequence elements, which could regulate the timing and place of gene expression during cellular differentiation and development.

In recent decades, because of the addition of the huge family of noncoding genes, RNAs have provoked great interest for their mysterious roles in organisms. lncRNA-encoded peptides expand the horizon of functional mechanisms for these bio-macromolecules. To date, thousands of peptide products have been identified in human cells, with limited understanding of their function. The current review has summarized the recently discovered micropeptides implicated in various biological processes. We also discussed the potential noncoding roles of mRNAs as a regulator. The continued discovery and functional characterization of bifunctional RNAs will provide new insights into important cellular processes and organismal evolution.

## Author Contributions

Both authors have made a substantial, direct and intellectual contribution to the work, and approved it for publication.

## Conflict of Interest Statement

The authors declare that the research was conducted in the absence of any commercial or financial relationships that could be construed as a potential conflict of interest.

## Abbreviations

4E-BP: Eukaryotic translation initiation factor 4E (eIF4E)-binding protein
aa: amino acid
CCR2: C-C chemokine receptor type 2
ceRNAs: competing endogenous RNAs
circRNAs: circular RNAs
FBXW7: F-Box and WD repeat domain containing 7
Foxo: Forkhead box-o
HIST1H1C: histone cluster 1 H1 family member C
hnRNPC: heterogeneous nuclear ribonucleoprotein C
hTR: human telomerase RNA
IRES: internal ribosome entry site
lncRNA: long non-coding RNA
Mbl: Mannose-binding lectin
Mdm2: mouse double minute 2
MLN: myoregulin
MOTS-C: mitochondrial open reading frame of the 12S rRNA-c
MS: mass spectrometry
nt: nucleotide
ribo-seq: ribosome profiling sequencing
Scl: sarcolamb
SERCA: sarco/endoplasmic reticulum Ca2+-ATPase
sORF: small open reading frame
SR: sarcoplasmic reticulum
SRA: steroid receptor RNA activator
SRAP: steroid receptor RNA activator protein
Ube3a1: ubiquitin-protein ligase E3A
UTRs: untranslated regions
VEGF: vascular endothelial growth factor
ZNF609: zinc Finger Protein 609
